# Percutaneous Lymphatic Drainage Through the Thoracic Duct: New Paths in Heart Failure

**DOI:** 10.1016/j.cjco.2023.04.008

**Published:** 2023-05-05

**Authors:** Vassili Panagides, François Côté, Abubaker Khalifa, Florence Bernier, Josep Rodes-Cabau, Mathieu Bernier

**Affiliations:** a Division of Cardiology, Quebec Heart & Lung Institute, Laval University, Quebec City, Quebec, Canada; b Division of Radiology, Quebec Heart & Lung Institute, Laval University, Quebec City, Quebec, Canada; c Division of Cardiology, Joseph Brant Hospital, McMaster University, Hamilton, Ontario, Canada

**The lymphatic system is intricately linked to clinical manifestations of heart failure. Unfortunately, this complex drainage network is commonly neglected in the pathophysiology and treatment of venous congestion and interstitial edema. The case reported here illustrates the safety and feasibility of percutaneous lymphatic drainage to treat clinical manifestations of heart failure.**

Edema occurs when an excessive volume of fluid accumulates in the tissues—either cells or interstitial space. Several factors can lead to interstitial edema, such as changes in hydrostatic or oncotic pressure, alterations of the molecular structure of the barrier (changes in hydraulic conductance or surface), and alterations in the lymphatic outflow system. Fluid leakage out of the capillaries to the interstitial space occurs physiologically for tissue hydration and nutrition. The lymphatic vascular system participates in interstitial volume homeostasis by scavenging liters of interstitial water and protein per day, and returning them to the venous circulation via lymphatic ducts located in the subclavian veins. Apart from its role in tissue fluid homeostasis, the lymphatic vascular system is a complex network involved in the transport of immune cells and antigens responsible for adaptive immunity.

The lymphatic system is commonly overlooked when considering the pathophysiology of heart failure (HF). Yet, most HF hospitalizations and symptoms are related to manifestations of venous congestion rather than low cardiac output. Numerous mechanisms contributing to interstitial fluid accumulation with the involvement of the lymphatic system have been described previously^1^; these include higher capillary hydrostatic pressure, decreased drainage (due to an increase in central vein pressure), impaired lymph vessel integrity/compliance, and maladaptive lymphangiogenesis. Hence, tissue congestion occurs when the lymphatic vascular system fails to compensate for the capillary hydrostatic pressure and increase filtration volume in the interstitial space, causing typical manifestations of HF.

Contemporary management of acute decompensated HF focuses mainly on venous decongestion via diuretic therapy. However, even if this therapy is useful, patients often develop diuretic resistance with no treatment options left.^2^ Moreover, despite aggressive diuresis, 40% of patients are still moderately congestive at discharge, with impaired outcomes.^3^ Lastly, worsening of renal function a few days after hospitalization is common, and it may limit the uptitration of HF drugs after a HF episode.^4^

To date, only 2 human studies, published in the 1960s, have investigated the feasibility and efficacy of surgical lymphatic drainage in HF.^5^^,^^6^ No other study targeting the lymphatic system in HF has been published since then. In light of recent interest on the topic,^1^ our study aimed to evaluate the safety and technical feasibility of percutaneous transcatheter cannulation and drainage of the thoracic duct in patients with chronic HF and fluid overload.

## Case

### Patient description

A 65-year-old woman, with a history of mitral valve replacement 5 years before and numerous episodes of HF with preserved ejection fraction, was referred to our centre for persisting dyspnea (New York Heart Association class III) and congestion, despite a high dose of furosemide (250 mg twice a day) and guideline-directed medical therapy, including a sodium-glucose cotransporter-2 (SGLT2) inhibitor. Transthoracic echocardiography revealed increased gradients on the mitral bioprosthesis (mean: 10 mm Hg, similar to the postoperative period), preserved left ventricular ejection fraction, and moderate-to-severe tricuspid regurgitation with moderately impaired right ventricular function. Considering the severity of the symptomatology with no other therapeutic options, the medical team proposed that she participate in this protocol. The study was approved by the ethics committee of the centre, and signed informed consent was obtained before the procedure.

### Study procedure

**Figure 1:**
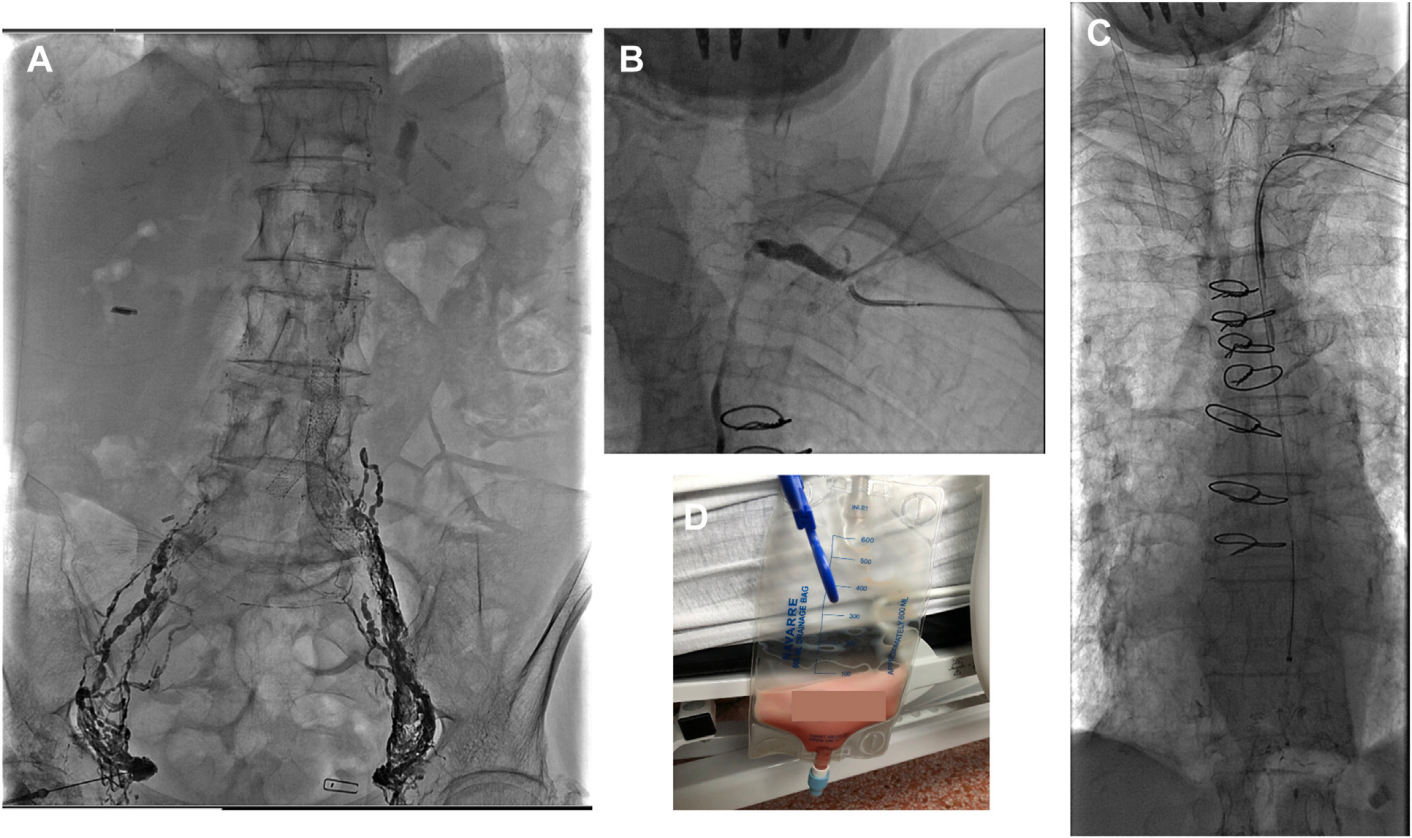
Description of the percutaneous lymph drainage of thoracic duct. (**A**) Intranodal lymphangiography after bilateral inguinal node puncture using a 22-gauge spinal needle. (**B**) Visualization of the thoracic duct. (**C**) Successful introduction of a 4-Fr introducer in the canal duct. (**D**) Lymph collection.

The intervention was performed with the patient under conscious sedation, using fluoroscopic guidance. The first step of the procedure consisted of an intranodal lymphangiography (Fig. 1A). This step was performed with an ultrasound-guided superficial bilateral inguinal node puncture using a 22-gauge spinal needle. After successful node puncture, a slow infusion (6 cc/hour) of Lipiodol (ethiodized oil; Guerbet, Princeton, NJ) through the needle led to a successful visualization of the lymphatic system and the thoracic duct (Fig. 1B). The humeral vein was then cannulated using a micro puncture kit followed by the insertion of 5-Fr Soft-Vu Navigate 3 angiographic catheter (Sos Omni Selective, AngioDynamics, Queensbury, NY). The thoracic duct was cannulated and crossed without puncture, using a 2.8-Fr PROGREAT microcatheter (Terumo Medical, Somerset, NJ) and a 45° angled 0.018ʹʹ GT Glidewire (Terumo). The Glidewire was exchanged for an SV-5 300-cm steerable guidewire (Cordis, Miami Lakes, FL), and a 4-Fr 90-cm Flexor Checkflo (Cook Medical, Bloomington, IN) introducer was introduced in the canal duct (Fig. 1C).

### Outcomes and follow-up

The day of the procedure, physical examination showed the presence of moderate peripheral edema with no ascites, and jugular venous pressure was estimated at 8 cm of water. A right catheterization examination showed a mean central venous pressure of 5 mm Hg, a mean pulmonary arterial pressure of 35 mm Hg, and a cardiac index of 2.2 L/min per m^2^. After cannulation, a waveform pressure curve in the canal duct was observed/analyzed, with a mean value of 80 mm Hg (simultaneous mean systemic pressure of 70 mm Hg). Given the observed pressures, the lymph was collected by gravity drainage only. After 24 hours, 1.5 L of lymph was retrieved (Fig. 1D). The right catheterization before discharge showed a decrease in pulmonary arterial pressure (mean: 15 mm Hg) and similar central venous pressure. Diuresis was maintained (1.6 L during the first 24 hours), but the levels of diuretics were lowered before discharge, to avoid hypovolemia. The canal duct pressures were remeasured before removal of the drain and were found to be much lower (mean: 10 mm Hg) than before drainage. Peripheral edemas were less significant, and jugular venous pressure was similar. The patient was discharged without any complication 48 hours after the procedure. Laboratory measurements before, during, and after drainage, and 1 month later, are presented in Table 1. No relevant or concerning variation of measured parameters was observed, confirming the safety of lymph removal. The N-terminal pro-B type natriuretic peptide (NT-pro-BNP) value at 8 hours was 1064 ng/L and decreased to 548 ng/L 2 days after the procedure. The weight also decreased from 44.5 kg to 42.9 kg. This decrease was transient, as 1 month after the procedure, the NT-pro-BNP levels and weight were similar to those at baseline. One month later, patient quality of life, as assessed by the Kansas City Cardiomyopathy Questionnaire, improved (from 34 points to 41 points), but the New York Heart Association class remained unchanged.

**Table 1:** Laboratory measurement before, during, and after drainage of the lymphatic duct

Variable	Pre-procedure	8 h after procedure	Day 1	Day 2	1 mo	Normal value or range
Weight, kg	44.5	—	42.9	—	47.3	**—**
Sodium, mmol/L	132	137	134	131	137	135–145
Potassium, mmol/L	3.1	4.0	4.2	5.5	3.7	3.3–5.3
Urea, mmol/L	5.9	9.4	11.5	8.6	3.9	1.7–8.5
Creatinine, μmol/L	59	65	68	57	45	40–90
NT-pro-BNP, ng/L	686	1064	733	548	819	< 285
Calcium, mmol/L	2.16	2.35	2.32	N/A	N/A	2.11–255
Albumin, g/L	31	31	26	N/A	N/A	35–50
Protein total, g/L	67	69	65	N/A	N/A	65–82
Hemoglobin, g/L	128	158	154	124	115	120–160
Hematocrit	0.392	0.501	0.490	0.397	0.364	0.370–0.470
Platelets, g/L	205	244	222	172	244	160–400
White cells, g/L	10.10	17	14.20	11.80	9.40	4.80–10.80
Lymphocytes, g/L	1.4	0.6	1.4	0.8	N/A	
Neutrophiles, g/L	7.7	15.30	11.6	9.9	N/A	—
Eosinophiles, g/L	0.007	0.0001	0.001	0.2	N/A	—
IgA, g/L	1.13	1.42	1.28	N/A	N/A	0.60–4.00
IgE, kg/UI per L	908	1100	997	N/A	N/A	< 100
IgG, g/L	8.64	10.19	8.38	N/A	N/A	7.00–16.00

N/A, not available; NT-pro BNP, N-terminal pro-B type natriuretic peptide.

## Discussion

HF is a syndrome characterized by high mortality incidence, frequent hospitalization, and reduced quality of life. Neuro-hormonal therapeutic agents and implantable devices are widely used to prevent HF hospitalization, reduce symptoms, and lower the incidence of mortality. However, management of chronic and acute HF congestion is limited to mainly diuretic therapy. This work highlights for the first time the feasibility of a new strategy to treat HF congestion—percutaneous cannulation and drainage of the lymph from the thoracic duct.

Two mechanisms are involved in the transport mechanism of lymph—intrinsic pumping (passive stretching and active contraction of smooth muscle cells, combined with the action of the unidirectional valves composing the collecting lymphatics) and extrinsic pumping (pressure resulting from surrounding tissues, vessels, and breathing). Namely, lymphatic pumping is a dynamic and highly regulated process and its failure can lead to interstitial edema. Lymph circulation deregulation in HF can be related to 2 factors—an impairment of lymph vessel integrity, and a reduction in lymph drainage related to high central venous pressure.

The hemodynamic stress of HF is known to be associated with systemic inflammation. Inflammation increases vascular permeability, and the leak of proteins into the interstitial space subsequently increases interstitial oncotic pressure. Consequently, an interstitial fluid accumulation occurs, necessitating an enhanced lymph flow. However, lymph transport also decreases in chronic inflammation (related to impaired vessel integrity and compliance), leading to maintained venous congestion.

High central venous pressure can also reduce the ability of the lymph to pour out into the venous circulation. Interesting to note is that the patient’s pressures measured in the canal duct were high (mean: 80 mm Hg), and were higher than the systemic pressures. This phenomenon may be related to the capacity of the lymph vasculature to increase lymph vessel contractility when outflow resistance occurs. Nevertheless, all these factors are hypothesis generating. This approach might play a role in congestive patients who are resistant to diuretics because of renal impairment, or patients with consequent peripheral edema and normovolemia related to an increase of vascular permeability.

This intervention appears to be safe, and the technique is relatively similar to the access used to manage thoracic duct leakage. Nevertheless, some aspects of the procedure need to be improved. First, the intranodal lymphangiography requires the patient to lie down for approximately 1 hour, which may not be possible in HF patients with orthopnea. Targeting axillary instead of inguinal lymph nodes may be a promising option. Second, lymphatic valvules are difficult to cross, and cannulation of the canal duct may be challenging. Third, the quantity of lymph that needs to be collected and the potential impact of this retrieval on the immune system are difficult to determine. An animal study using a dedicated device to enhance lymph flow without withdrawing any fluid has been published recently and illustrates other alternatives in this field.^7^ Finally, albumin and other plasma proteins were slightly affected by the drainage in this patient, but this one case study cannot allow us to draw any conclusion about the potential harm of this loss. An important point to underline is that the hemodynamic results of the procedure seemed transient, as the biomarker and weight levels were higher at 1-month follow-up. Therefore, this procedure might be suitable for only bailout congestive states with no other issues. Moreover, the results of this single case study should be interpreted with caution; further cases and studies are warranted to draw definitive conclusions.

## Conclusion

Transcatheter percutaneous drainage of lymph appears to be a feasible and promising technique to improve congestion and interstitial edema in HF patients.

**Novel Teaching Points**

• This study aimed to evaluate the feasibility and safety of percutaneous cannulation and drainage of the lymphatic canal duct to treat venous congestion of patients with chronic HF.
• This strategy was safe, and it improved biologic markers shortly after the procedure.
• Further studies are warranted to confirm this interesting new path.
